# Supplementary material for: Chronic low back pain causal risk factors identified by Mendelian randomization: a cross-sectional cohort analysis
Source: Spine J. Author manuscript; Available in PMC 2026 Aug 15. (PMC13477212; doi:10.1016/j.spinee.2024.12.029)
Supplement: Supplementary file 1 [file NIHMS2170225-supplement-1.docx]

**Appendix A: Study measurement details**

Outcome measure

The **PEG score** ranges from 0-10, with higher values indicating more pain interference. It is calculated as the mean of responses to 3 items:

1) Average pain in past week (0=no pain, 10=worst imaginable pain)
2) How much pain interfered with enjoyment of life in past week (0=did not interfere, 10=completely interfered)
3) How much pain interfered with general activity in past week (0=did not interfere, 10=completely interfered)

Exposures

The **PROMIS sleep disturbance** 6a t-score (range 31.7-76.1, higher=more sleep disturbance) was based on responses to the following 6 questions:

1) In the past 7 days, my sleep quality was: 1 (=very good) to 5 (=very poor)
2) In the past 7 days, my sleep was refreshing: 1 (=very much) to 5 (=not at all)

Responses to 3-6 ranged from 1 (=not at all) to 5 (=very much)

In the past 7 days:

3) I had a problem with my sleep.
4) I had difficulty falling asleep.
5) My sleep was restless.
6) I tried hard to get to sleep.

The responses to these questions were summed to produce a raw summary score (range 6-30), which was then mapped to a t-score, with 50 representing the mean of a reference population and 10 being the SD of that population.

The **PROMIS depression** 4a t-score (range 41.0-79.4, higher=more depressed) was based on responses to the following 4 questions, with responses ranging from 1 (=never) to 5 (=always): In the past 7 days…

1) I felt worthless.
2) I felt helpless.
3) I felt depressed.
4) I felt hopeless.

The responses to these questions were summed to produce a raw summary score (range 4-20), which was then mapped to a t-score, with 50 representing the mean of a reference population and 10 being the SD of that population.

**Alcohol use** was defined as number of drinks per week as a continuous variable in response to the question: How many alcoholic drinks do you consume per week, on average?

**Smoking** was measured as a categorical variable in reponse to the question: How would you describe your cigarette smoking?

1) Never smoked
2) Current smoker
3) Used to smoke, but have now quit

**Body mass index (BMI)** was calculated from self-reported weight and height. Obesity was defined by BMI of 30.0 kg/m^2^ or higher. Furthermore, underweight was defined as <18.5 kg/m^2^, normal weight ranges from 18.5-24.9 kg/m^2^, overweight ranges from 25-29.9 kg/m^2^, obesity class I ranges from 30-34.9 kg/m^2^, obesity class II ranges from 35-39.9 kg/m^2^, and obesity class III constitutes ≥40 kg/m^2^.

Type A confounding variables

**Age** was measured as a continuous variable and **sex** as a binary variable (male/female). Categories for **education** were some high school, high school completed, associates/technical degree completed, college/baccalaureate degree completed, doctoral/postgraduate education. Categories for **relationship status** were married, never married, divorced, domestic partner, widowed, separated. Participants were asked how difficult it was to pay for basic necessities; “hard” and “very hard” responses were classified as high **financial strain**.

The **PROMIS anxiety 4a T-score** (range:40.3-81.6, higher=more anxiety) was based on responses to the following 4 questions, with responses ranging from 1 (=never) to 5 (=always): In the past 7 days…

1) I felt fearful
2) I found it hard to focus on anything other than my anxiety
3) My worries overwhelmed me
4) I felt uneasy

The responses to these questions were summed to produce a raw summary score (range 4-20), which was then mapped to a t-score, with 50 representing the mean of a reference population and 10 being the SD of that population.

The **Fear Avoidance score** (FABQ-PA) (58) (range 0-24, higher=more avoidance) was calculated as the sum of responses (0=completely disagree, 6=completely agree) to the following 4 items:

1) Physical activity makes my pain worse.
2) Physical activity might harm my back.
3) I should not do physical activities which might make my pain worse.
4) I cannot do physical activities which might make my pain worse.

The **Pain Catastrophizing Scale** SF (PCS-6) (59) (range 0-12, higher=more catastrophizing) included 3 subscales -- Helplessness, Magnification, and Rumination -- with each subscale having 2 components. The responses to the following 6 statements ranged from 0=not at all to 4=all the time:

When I’m in pain …

1) It’s awful and I feel that it overwhelms me. (Helplessness subscale)
2) I feel I can’t stand it anymore. (Helplessness subscale)
3) I become afraid that the pain will get worse. (Magnification subscale)
4) I wonder whether something serious may happen. (Magnification subscale)
5) I keep thinking about how much it hurts. (Rumination subscale)
6) I keep thinking about how badly I want the pain to stop. (Rumination subscale)

The mean for each subscale was determined, resulting in a 0-4 score. The 3 subscales were then summed to create the total score, ranging from 0-12, with higher scores representing more pain catastrophizing.

Type B confounding variables

**Current opioid use** was defined as current use for low-back pain or current daily use for any reason. **Expectation of pain relief** over next 3 months was assessed by the question: Please indicate how much pain relief you expect over the coming three months (range 1 = no relief, and 10 = complete relief). **Duration of low back pain** was recorded as 3-6 months, 6 months -1 year, 1-5 years or more than 5 years. **History of low-back operation** was assessed and recorded as none, decompression surgery or spinal fusion surgery. **Discrimination** was assessed by the question: How often do people treat you unfairly because of your ethnicity or race? 1= never, 2 = sometimes, 3 = often, 4 = always.

**Post traumatic stress disorder (PTSD)** was noted if participant marked “yes” to having experienced things as a child or as an adult that are unusually or especially frightening, horrible, or traumatic (examples include a serious accident or fire, a physical or sexual assault or abuse, an earthquake or flood, a war, seeing someone be killed or seriously injured, having a loved one die through homicide or suicide) AND participant reported at least 3 of 5 symptoms in past month (nightmares, avoided triggering situations, on guard, felt detached, self-blamed).

Those who marked “yes” to “have you filed or been awarded a worker’s compensation claim related to your back problem,” “are you involved in a lawsuit or legal claim related to your back problem,” or “have you ever applied for, or received, disability insurance for your pain condition” were marked as having filed **workers compensation, lawsuit, or disability due to back problem or pain**.

**Pain self-efficacy score** (PSEQ-4) (60) was calculated as the sum of responses to 4 questions on how confident the participant was in doing the following (0=not at all confident, 6=completely confident):

1) I can cope with my pain in most situations.
2) I can still do many of the things I enjoy doing, such as hobbies or leisure activity, despite the pain.
3) I can still accomplish most of my goals in life, despite the pain.
4) I can live a normal lifestyle, despite the pain.

The total score ranged from 0-24, with higher scores representing greater confidence.

T-scores were calculated for the following PROMIS measures: **cognitive function** 2a, **fatigue** 4a, and **social isolation** 4a (41).

**Appendix B: ESC-DAG decision log**

Decision Log Type A confounders

| Edge | Level of Evidence^a^ | References |
| --- | --- | --- |
| Anxiety -> Comorbidity (Charlson Comorbidity index (CCI)) | 5 | (61-64) |
| Depression -> Comorbidity (CCI) | 5 | (61-69) |
| Sleep -> Comorbidity (CCI) | 5 | (70-73) |
| Financial strain -> Obesity | 3 | (61, 74-76) |
| Obesity -> Comorbidity (CCI) | 5 | (61, 77) |
| Obesity -> Depression | 5 | (61) |
| Obesity -> Anxiety | 5 | (61, 77, 78) |
| Obesity -> PEG | 5 | (10, 33, 61) |
| Anxiety -> Sleep | 5 | (73, 79, 80) |
| Anxiety -> Smoking | 5 | (79, 81, 82) |
| Depression -> Smoking | 5 | (79, 81, 82) |
| Sleep -> Depression | 5 | (73, 79, 83) |
| Depression -> PEG | 5 | (31, 38, 61, 84, 85) |
| Anxiety -> PEG | 5 | (10, 61, 85) |
| Sleep -> PEG | 5 | (25, 31, 79) |
| Education -> Comorbidity (CCI) | 5 | (86-90) |
| Education -> Obesity | 5 | (90, 91) |
| Education -> Anxiety | 5 | (64, 92) |
| Education -> Depression | 5 | (64, 79, 90, 92, 93) |
| Education -> Financial stress | 3 | (94, 95) |
| Education -> Smoking | 5 | (84, 96, 97) |
| Education -> PEG | 5 | (38, 79, 98, 99) |
| Financial strain -> Anxiety | 3 | (100, 101) |
| Financial strain -> Depression | 3 | (100-102) |
| Financial strain -> Sleep | 2 | (103, 104) |
| Financial strain -> PEG | 2 | (79, 98, 105) |
| Sleep -> Smoking | 5 | (73, 79) |
| Smoking -> PEG | 5 | (24, 38, 99) |
| Catastrophization -> Anxiety | 2 | (106, 107) |
| Catastrophization -> Depression | 2 | (106, 107) |
| Catastrophization -> PEG | 4 | (85, 106, 108-112) |
| Fear Avoidance -> Anxiety | 2 | (106, 107, 113) |
| Fear Avoidance -> Depression | 2 | (106, 107, 113) |
| Fear Avoidance -> PEG | 4 | (85, 106, 110-112) |
| Age -> Comorbidity (CCI) | 2 | (61, 114) |
| Age -> Obesity | 3 | (61, 115) |
| Age -> Sleep | 3 | (79, 116) |
| Age -> PEG | 4 | (61, 79, 106, 117, 118) |
| Age -> Smoking | 3 | (79, 119) |
| Age -> Anxiety | 3 | (79, 106, 120-123) |
| Age -> Depression | 3 | (79, 106, 120, 124-126) |
| Age -> Catastrophization | 2 | (106, 113, 127) |
| Age -> Fear Avoidance | 2 | (106, 113, 128) |
| Age -> Financial Strain | 2 | (129) |
| Sex -> Comorbidity (CCI) | 5 | (61, 130) |
| Sex -> Obesity | 5 | (61, 130) |
| Sex -> Sleep | 3 | (131) |
| Sex -> PEG | 4 | (61, 99, 132) |
| Sex -> Smoking | 3 | (133) |
| Sex -> Anxiety | 3 | (61, 134) |
| Sex -> Depression | 3 | (61, 134) |
| Sex -> Catastrophization | 3 | (106, 127, 132) |
| Sex -> Fear Avoidance | 3 | (106, 135) |
| Sex -> Financial strain | 4 | (136) |
| Sex -> Education | 3 | (106, 137, 138) |
| Alcohol -> Sleep | 5 | (139) |
| Anxiety -> Alcohol | 5 | (140-142) |
| Depression -> Alcohol | 5 | (143-146) |
| Age -> Alcohol | 5 | (147-149) |
| Sex -> Alcohol | 5 | (149) |
| Alcohol -> Financial strain | 5 | (145) |
| Education -> Alcohol | 3 | (150) |
| Alcohol -> Comorbidity (CCI) | 3 | (149, 151) |
| Alcohol -> Obesity | 5 | (152) |

^a^Levels of evidence:

Level 1- Expert opinion only (based on causal models published in the literature)

Level 2- Cross-sectional assocation

Level 3- Temporal order (longitudinal studies demonstrating that the exposure precedes cLBP)

Level 4- Analysis-based confounding control

Level 5- Design based confounding control (e.g. MR, twin studies)

Decision Log Type B confounders

| **Confounder** | **Exposures** | **References** |
| --- | --- | --- |
| Expectations | Obesity | (153-158) |
| Self Efficacy | Obesity, Alcohol, Smoking | (85, 111, 159-165) |
| PTSD | Depression, Obesity, Sleep, Smoking, Alcohol | (166-172) |
| Compensation | Obesity, Smoking, Alcohol | (173-183) |
| Mistreatment | Depression, Obesity, Sleep, Smoking, Alcohol | (184-191) |
| Relationship status | Depression, Obesity, Sleep, Smoking, Alcohol | (192-197) |
| Surgery | Depression, Obesity, Sleep, Smoking, Alcohol | (198-205) |
| Opioid use | Depression, Obesity, Sleep, Smoking, Alcohol | (206-211) |
| Duration of LBP | Depression, Obesity, Sleep, Smoking, Alcohol | (58, 173, 212-220) |
| Isolation | Depression, Sleep, Alcohol | (221-225) |
| Fatigue | Depression, Obesity, Sleep, Smoking, Alcohol | (226-234) |
| Cognitive Function | Depression, Obesity, Sleep, Smoking, Alcohol | (235-240) |
